# Supplementary material for: The control costs of human brain dynamics
Source: Netw Neurosci. 2025 Mar 3;9(1):77–99. doi: 10.1162/netn_a_00425 (PMC11949579; doi:10.1162/netn_a_00425)
Supplement: Supplementary file 1 [file netn-9-1-77-s001.pdf]

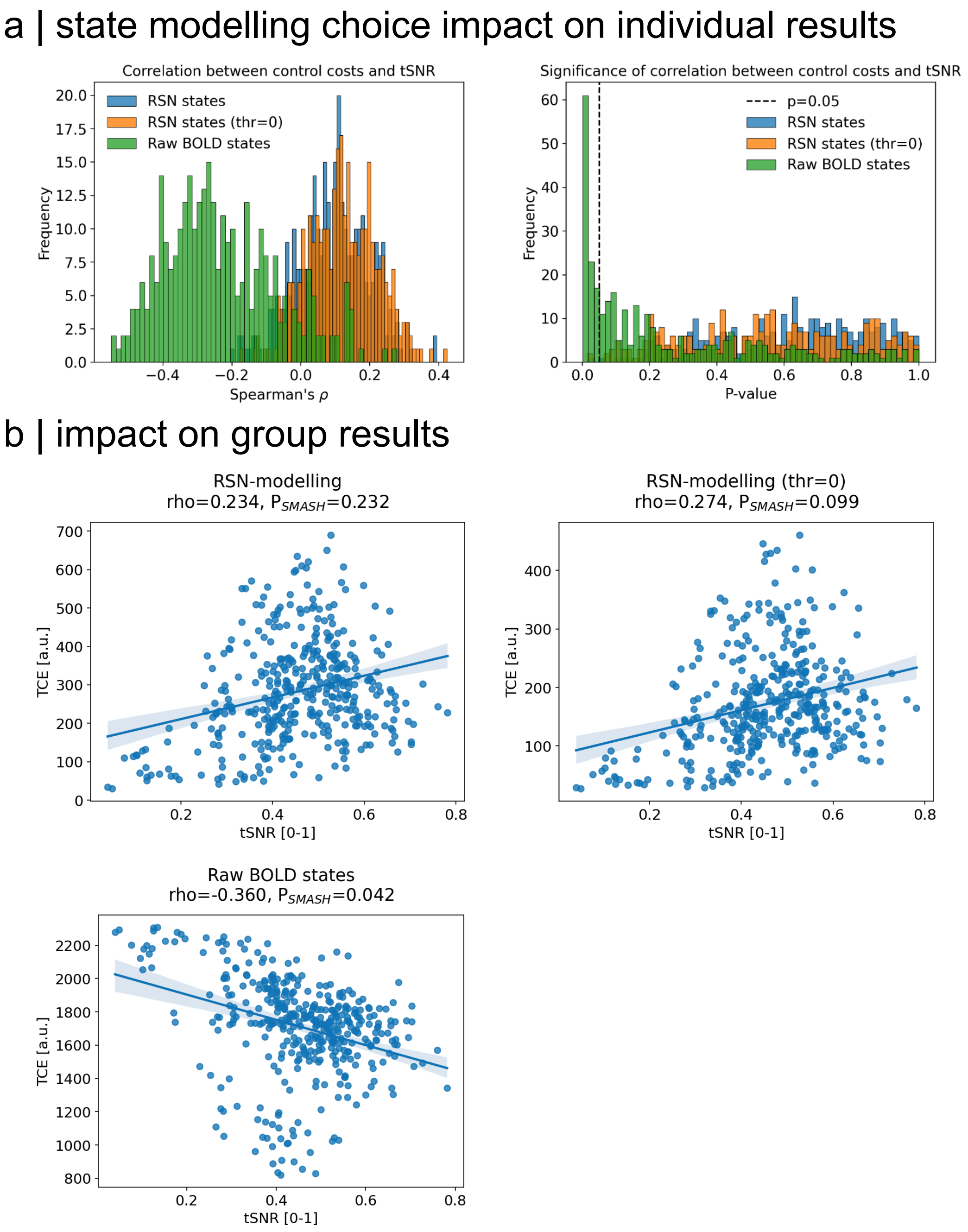

Figure S1. **Intrinsic network modelling validation |** (a) The temporal signal-to-noise ratio (tSNR) of each individual's recording was estimated by dividing their mean signal prior to demeaning with their signal variability [95]. This metric was compared to their control costs from three modelling approaches: 1) our reported approach to use intrinsic networks as states, where only time points with an average network activity above 0.5 are selected; 2) same as 1) but with an activity threshold of 0; 3) raw BOLD activity are used as states. The first two approaches result in TCE maps that correlate similarly to their respective tSNR (left), with only few subjects showing significance in their associations(right). Using the third approach, however, results in control costs being negatively correlated to tSNR (left), and more individual's control maps being significantly related to the tSNR of their recording (right). (b) We see similar results on a group-level, where we average all control maps and tSNR across subjects. In both state modelling approaches, we observe that control costs and tSNR are not significantly associated, however, the lower threshold variant results are statistically more unlikely to be discernible [Spearman ρ = 0.234, P_{SMASH} = 0.232 for thr=0.5 and Spearman ρ = 0.274, P_{SMASH} = 0.099 for thr=0]. Critically, the group-average map of TCE significantly correlates with tSNR when opting for no state modelling [Spearman ρ = -0.36, P_{SMASH} = 0.042].

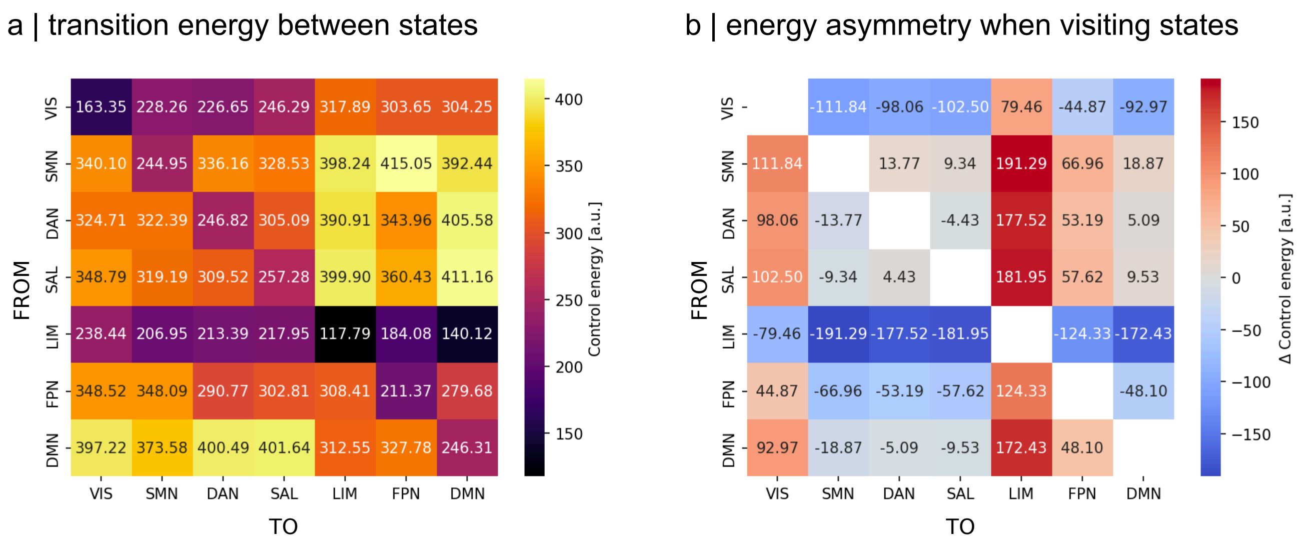

Figure S2. **Transition energy matrix |** (a) We simulate the control energy to transition between all of pairs of intrinsic networks. Similar to previous work [49, 64], we find that the costs to transition into a state are dissimilar to the costs of leaving it. (b) We highlight this asymmetry by subtracting the transition energy matrix with its transpose, thereby accentuating the difference between going into and leaving a state.

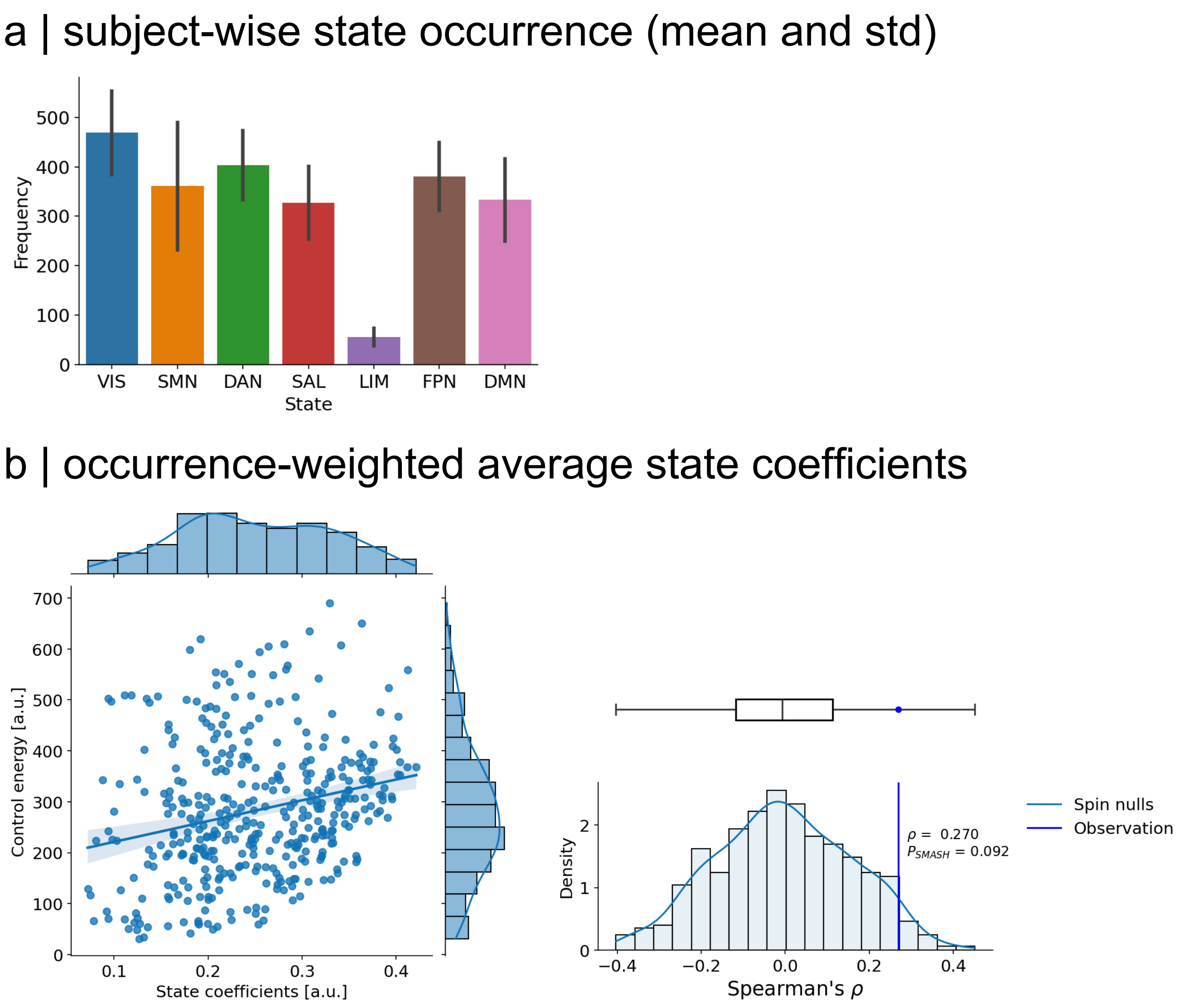

Figure S3. **Control costs vs. state coefficients** | (a) State occurrences are counted for each individual. We report the average and standard deviation of each state distribution. (b) Left: Weighting each network map by their occurrence and then summing all maps yields an occurrence-weighted state coefficient map. This is averaged across all individuals and compared to the group-level TCE map. Right: TCE and state coefficients are not significantly correlated when accounting for spatial autocorrelation [Spearman $\rho = 0.27$, $P_{\mathrm{SMASH}} = 0.092$].

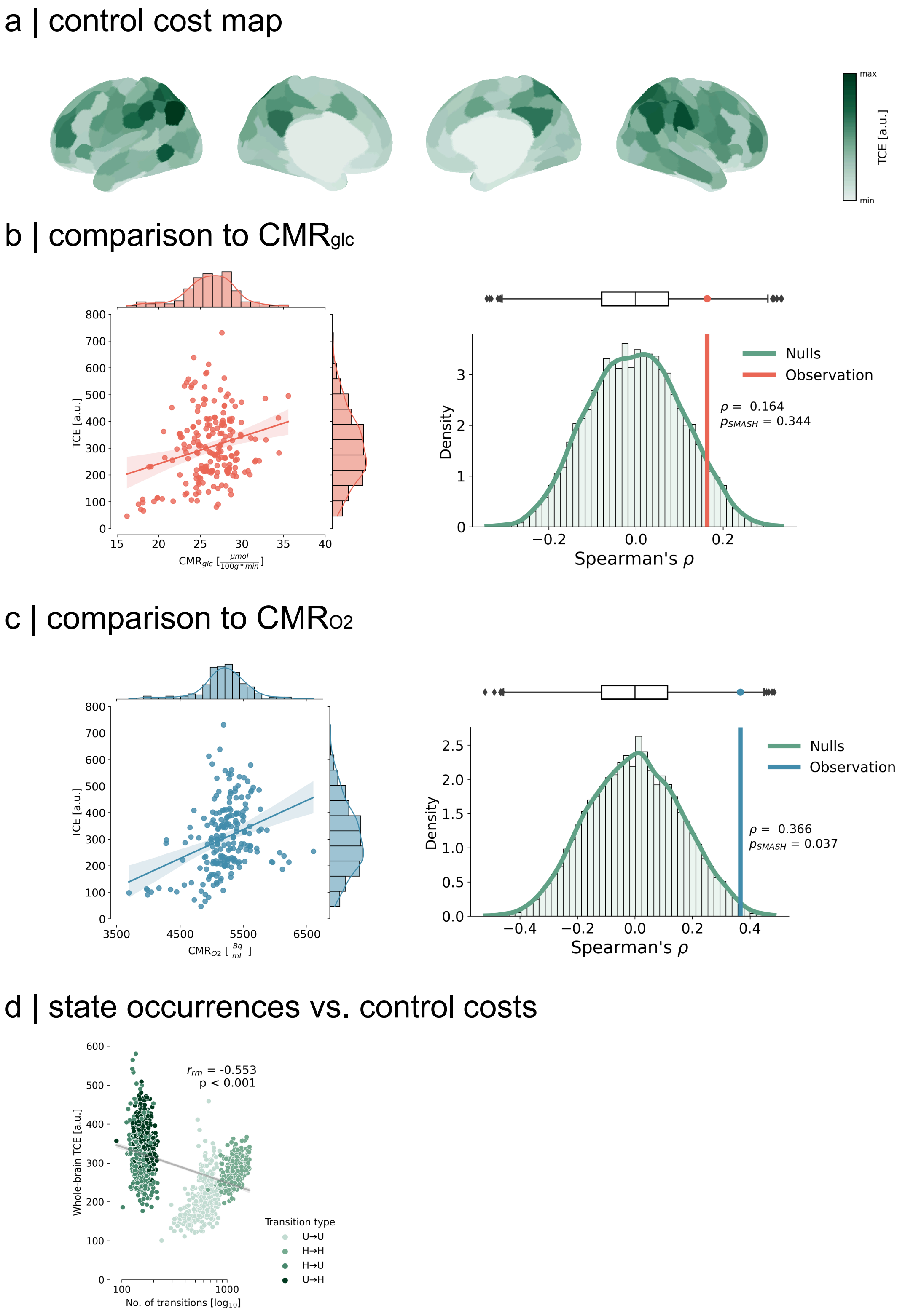

Figure S4. **Replication with Schaefer 200 parcellation|** (a) TCE map across both hemispheres. (b) No significant relationship to CMR_{glc} [Spearman ρ = 0.164, P_{SMASH} = 0.344]. (c) Significant relationship to CMR_{O2} [Spearman ρ = 0.366, P_{SMASH} = 0.037]. (d) Whole-brain TCE is inversely related to the number of transitions across hierarchies [r_{rm} = -0.553, P < 0.001].

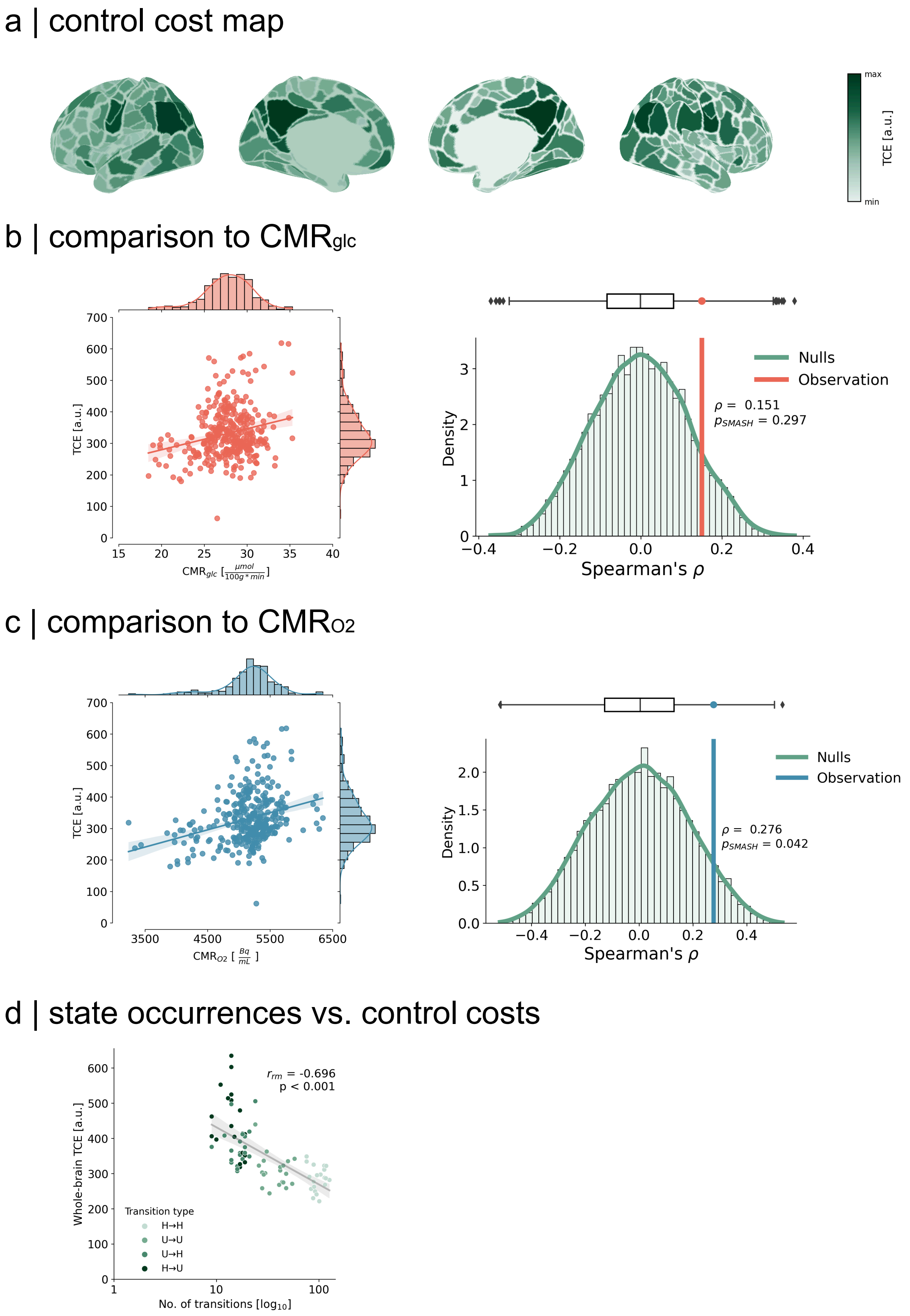

Figure S5. **Replication with Gordon parcellation |** (a) TCE map across both hemispheres. (b) No significant relationship to CMR_{glc} [Spearman ρ = 0.151, P_{SMASH} = 0.297]. (c) Significant relationship to CMR_{O2} [Spearman ρ = 0.276, P_{SMASH} = 0.042]. (d) Whole-brain TCE is inversely related to the number of transitions across hierarchies [r_{rm} = -0.696, P < 0.001].

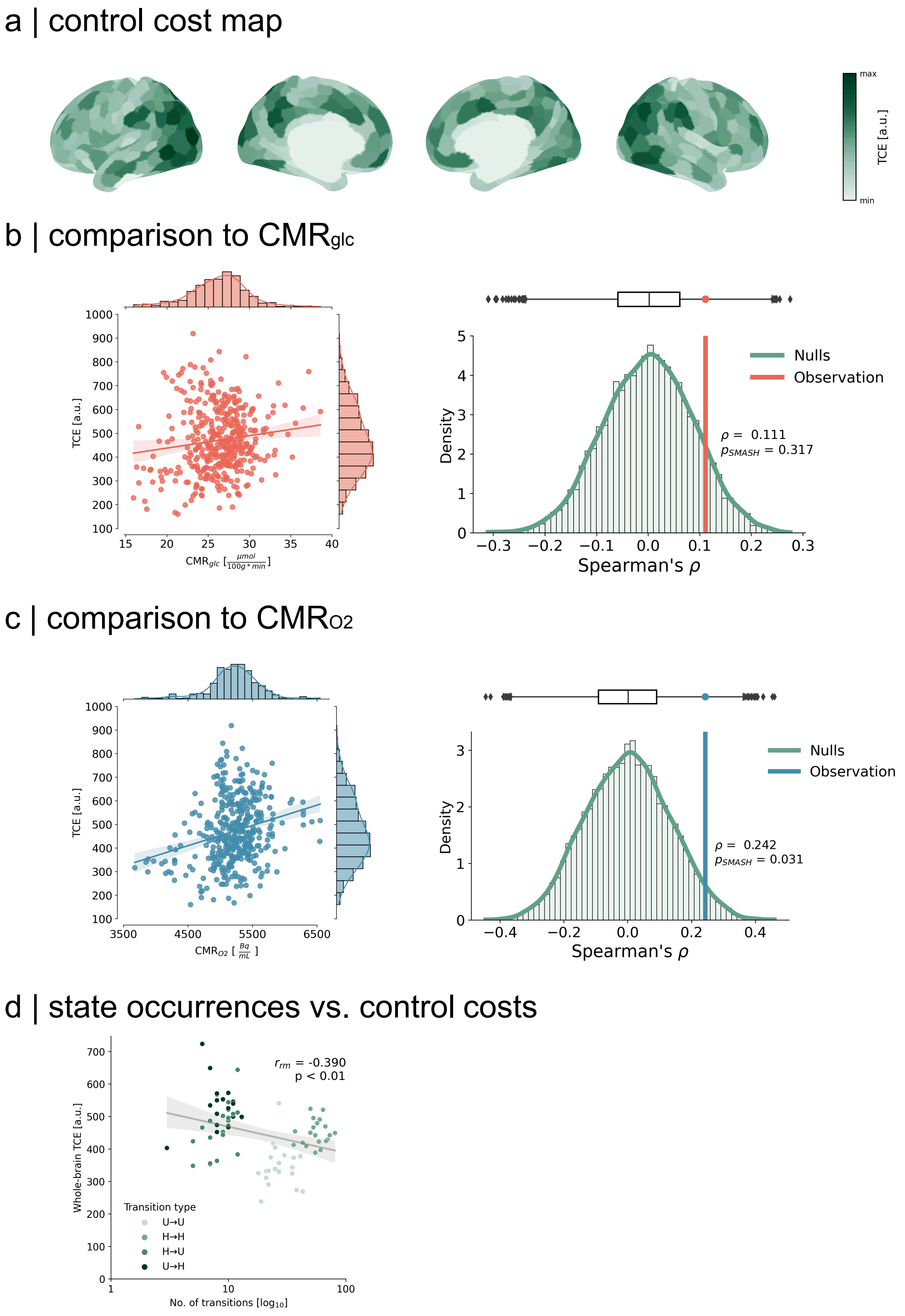

Figure S6. **Replication with separate dataset [15]** | (a) TCE map across both hemispheres. (b) No significant relationship to CMR_{glc} [Spearman ρ = 0.111, P_{SMASH} = 0.317]. (c) Significant relationship to CMR_{O2} [Spearman ρ = 0.242, P_{SMASH} = 0.031]. (d) Whole-brain TCE is inversely related to the number of transitions across hierarchies [r_{rm} = -0.39, P $<$ 0.01].
